# Prevalence of sexual dysfunction in male subjects with alcohol dependence

**DOI:** 10.4103/0019-5545.33257

**Published:** 2007

**Authors:** Bijil Simon Arackal, Vivek Benegal

**Affiliations:** Deaddiction Centre, National Institute of Mental Health and Neurosciences, Bangalore - 560 029, India

**Keywords:** Alcohol dependence, sexual dysfunction

## Abstract

**Background:**

Chronic and persistent alcohol use is known to induce sexual dysfunction, which leads to marked distress and interpersonal difficulty.

**Aim:**

We attempted to assess the prevalence of sexual dysfunction in a clinical sample of subjects with alcohol dependence.

**Materials and Methods:**

One hundred male subjects admitted to a deaddiction centre with a diagnosis of alcohol dependence syndrome with simple withdrawal symptoms (F10.30, ICD-10 criteria) were assessed for sexual dysfunction using a sexual dysfunction checklist, constructed using items from the Diagnostic Criteria for Research [ICD-10] for sexual dysfunction.

**Results:**

Seventy-two per cent had one or more sexual dysfunction, the most common being premature ejaculation, low sexual desire and erectile dysfunction. The amount of alcohol consumed appeared to be the most significant predictor of developing sexual dysfunction.

**Conclusion:**

Sexual dysfunction is common in patients with alcohol dependence. Heavy drinking proportionately increases the risk. Clinicians need to routinely assess sexual functioning in alcoholic patients so that other factors contributing to sexual dysfunction can be ruled out.

Chronic and persistent alcohol use is known to induce sexual dysfunction, which leads to marked distress and interpersonal difficulty. This, in turn, is known to worsen the alcohol abuse. Sexual dysfunction in the alcoholic may be due to the depressant effect of alcohol itself, alcohol-related disease or due to a multitude of psychological forces related to the alcohol use.[1] The spectrum of sexual dysfunction encompasses:

1. Decreased sexual desire—persistent or recurrent deficiency or absence of desire for sexual activity giving rise to marked distress and interpersonal difficulty;
2. Sexual aversion disorder—persistent or recurrent aversion and avoidance of all genital sexual contact leading to marked distress and interpersonal difficulty;
3. Difficulty in erection—recurrent or persistent, partial or complete failure to attain or maintain an erection until the completion of the sex act;
4. Difficulty in achieving orgasm—persistent or recurrent delay in or absence of orgasm, following a normal sexual excitement phase;
5. Premature ejaculation—persistent or recurrent ejaculation with minimal sexual stimulation, before, on or shortly after penetration and before the person wishes it, which causes marked distress.[2]

Alcohol abuse is the leading cause of impotence and other disturbances in sexual dysfunction.[3] Episodic erectile failure in alcoholic men is fairly routine, found to be significantly higher in men consuming more than three standard units of alcohol (12 g ethanol) daily and in subjects smoking more than 10 cigarettes/day.[4] Van Thiel and Lester[5] reported that 61% of patients dependent on alcohol reported sexual dysfunction, the most common being erectile dysfunction followed by reduced sexual desire. Erectile dysfunction and reduced sexual desire were frequently seen to be coexisting.[6–9] Vijayasenan,[10] found that of 97 male inpatients admitted for the treatment of alcoholism, 71% suffered from sexual dysfunction for a period of more than 12 months prior to admission to a hospital. The disturbances noted were diminished sexual desire (58%), ejaculatory incompetence (22%), erectile impotence (16%) and premature ejaculation (4%). Virtually all aspects of the human sexual response are affected by alcohol especially sexual desire and erection.[11]

Schiavi *et al.*[12] failed to find any difference in sexual dysfunction in alcoholics abstinent for 2-3 months in comparison with a nonalcoholic control group, speculating that alcohol-induced sexual dysfunction was reversible with abstinence. The aim of the present study was to estimate the prevalence of sexual dysfunction in males with alcohol dependence. We specifically assessed male subjects admitted to a treatment center with a diagnosis of alcohol dependence syndrome, without obvious hepatic cirrhosis or other co-morbidity. Female patients were excluded from the study as the number of women who use alcohol in India are few and the number of female alcoholics who avail of treatment centers are too few to contribute to significant statistical power. Also, the spectrum of sexual dysfunction is different in the female from the male.

## MATERIALS AND METHODS

One hundred male subjects, consecutively admitted to the Deaddiction Centre of the National Institute of Mental Health And NeuroSciences (NIMHANS), Bangalore, India, with a diagnosis of Alcohol Dependence Syndrome With Simple Withdrawal Symptoms (F10.30, ICD-10 criteria) [WHO][13] were recruited for the study. All subjects gave informed consent for taking part in the study. Subjects were initially assessed on the schedules for clinical assessment in neuropsychiatry (SCAN)[14] by a trained psychiatrist (VB). All patients were subjected to detailed clinical and biochemical examinations including blood glucose and liver enzymes. Patients with significantly high levels of liver enzymes or physical findings suggestive of hepatic cirrhosis were referred for ultrasound assessment of the abdomen.

Subjects were included if they were:

- between 20-50 years of age
- married or had a regular sexual partner

Patients were excluded if they had a

- Clinically assessed history of primary sexual dysfunction [prior to initiation of alcohol use]
- Co-morbid physical disorders: diabetes mellitus, hypertension, signs and symptoms suggestive of alcoholic cirrhosis, a clinical diagnosis of endocrine disorders, other systemic illnesses, history of genito-urinary surgery and neurological or spinal cord lesions.
- Co-morbid psychiatric disorders: schizophrenia, delusional disorder, anxiety disorders and mood disorders including dysthymia. Patients who had symptoms of depression or anxiety not fulfilling a syndromal diagnosis were included in the study.
- Substance use other than alcohol and tobacco.
- Use of drugs affecting sexual function (antipsychotics, antidepressants, antihypertensives, steroids, disulfiram etc.)

All the above subjects were assessed for the prevalence of one or more sexual dysfunction experienced over the past 12 months using a sexual dysfunction checklist (Appendix A) by a trained psychiatrist (BSA). The checklist contains items corresponding to 12 areas of sexual dysfunction described in the Diagnostic Criteria for Research, ICD-10 Classification of Mental and Behavioural Disorders.[15] This was necessary as the SCAN does not contain a detailed assessment for the ICD-10 section on Sexual dysfunction not caused by organic disorder or disease (F52). The disorders specifically tapped by the checklist were aversion towards sex, low sexual desire, difficulty in achieving and in maintaining erection, premature ejaculation, inhibited or delayed ejaculation orgasm with flaccid penis, anorgasmia, pain at the time of coitus, dissatisfaction with frequency of intercourse per week (in the last year and in a representative week 5 years earlier), partner and, own sexual function.

Sexual dysfunction was rated for the last one year and temporary or situational complaints were ignored. Data regarding the quantity of alcohol usually consumed per day [in standard drinks; where 1 drink = 30 ml. Spirits = 330 ml. Beer = 1/3 sachet of arrack] and duration of dependence, was extracted from the items corresponding to the section on Mental and Behavioural disorders due to use of alcohol [F10.0] in the SCAN and used in the analyses. However, only the presence or absence of tobacco consumption and not a measure of severity was used for analyses. The ratings were sought after two weeks of inpatient stay after the period of detoxification with benzodiazepines.

## RESULTS

The 100 male subjects had a mean age of 37.09 (± 6.74) years. The quantity of alcohol consumed per day was 20.6 (± 9.07) standard drinks [8-42 drinks per day]. The mean duration of alcohol dependence was 8.59 (± 6.64) years. 87% of the subjects also used tobacco [chewing and / or smoking]. Seventy-two of the 100 subjects reported one or more sexual dysfunction. Four (4%) subjects reported aversion to sex to the extent that they had not attempted sexual intercourse in the last one year. Consequently, the prevalence of sexual dysfunction other than aversion to sex and low sexual desire, had to be calculated after excluding these 4 subjects.

Premature ejaculation was reported by 36 out of 96 (37.5%) subjects, out of which, 27 (28.12%) had complaints of ejaculating within the first minute itself and the rest (9.38%) ejaculated within three minutes of intromission. The next most frequent sexual dysfunction reported was low sexual desire, which was reported by 36 out of 100 subjects. Erectile dysfunction was reported by 33.3% of the subjects with difficulty in achieving erection in 19 subjects (19.79%) and difficulty in maintaining erection in 13 subjects (13.54%).

Fourteen subjects (14.58%) had a lack of pleasure at the time of ejaculation (anorgasmia) and 10 (10.41%) had inhibited or delayed ejaculation. Next was the complaint of dissatisfaction with the frequency of sexual intercourse in 26 people (27.03%) and dissatisfaction with own sexual function reported by 19 patients (19.79%).

Nine subjects (9.37%) had dissatisfaction with the sexual relationship with their partner and eight subjects reported (8.33%) orgasm with flaccid penis. Coital pain or feeling of pain in genitals at the time of sexual intercourse was seen in six subjects (6.1%).

There was a significant reduction in the frequency of sexual intercourse per week over the last five years having decreased from a mean of 4.6 (± 2.6) times per week to 2.2 (± 2.2) times per week currently. Forty-eight per cent of the sample had more than one sexual dysfunction. Of the 24 subjects with only one complaint, the most frequent complaint was that of premature ejaculation in 18 subjects.

**Figure 1:**
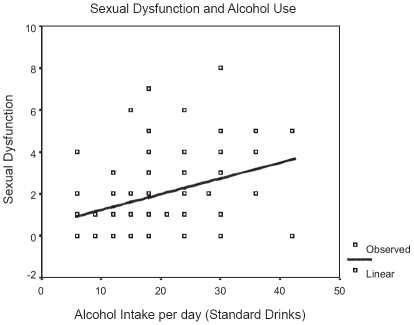
Linear regression illustrates the predictive relationship between the amount of alcohol consumed and the likelihood of any sexual dysfunction.

The number of sexual dysfunction complaints was significantly associated with the amount of alcohol consumed per day. On curve-fitting the data, there was a significant positive linear relationship (F = 10.54; dF 87; *P* = 0.002) [Figure 1]. However, there was no correlation between the reduction in frequency of sexual intercourse over the last five years and the amount of alcohol consumed.

There appeared to be no significant correlations between the subjects' ages and duration of alcohol dependence with the number of complaints of dysfunction. People with tobacco use were no more likely to have more sexual dysfunction than those without tobacco use [t = -1.32; dF 97; *P* = 0.19].

## DISCUSSION

Sexual dysfunction appears to be common among male subjects with alcohol dependence. Seventy-two per cent of the subjects with alcohol dependence complained of one or more problems with sexual functioning. This is similar to what has been reported in earlier studies.[10,16] Multiple co-existing dysfunctions seemed to be the norm in the sample studied. The most common condition reported in our study was premature ejaculation followed closely by low sexual desire and erectile dysfunction.

The number of symptoms reported appeared to be a function of the amount of alcoholic beverage consumed. The chance of developing sexual dysfunctions appears to increase with increasing quantity of alcohol consumed. Higher levels of alcohol intake may result in greater neurotoxic effects. It has been reported that heavy alcohol use may contribute to a reversible vagal neuropathy, which is perhaps reversible on abstinence.[17] However, chronic heavy use of alcohol is also known to significantly alter gonadal hormones.[9]

There is also a significant population, which has psychogenic sexual dysfunction, which is likely in a situation of marital conflict, which commonly exists in the families of alcoholics.[18] There is some evidence of this with more than a third of the subjects reporting dissatisfaction with their spouses' responses and / or decreased frequency. This cannot be conclusive without data on nocturnal erection or sexual activity in alternate situations. One of the limitations of this exploratory study is that marital functioning was not specifically assessed.

Counterintuitively, the likelihood of developing sexual dysfunction did not depend on the number of years of alcohol dependence or on the age of the subject. One reason for these findings may be the narrow range of ages at presentation and durations of dependence across the group.

Tobacco use though, was not found to be a significant determinant of sexual dysfunction. This is contrary to all reported evidence.[19] This finding is most likely to be due to our treatment of tobacco use as a categorical (present / absent) variable in a situation where almost 90% of the sample was using tobacco. Future studies need to use indices of severity to avoid this error.

The exclusive focus on male alcoholics was necessitated by the fact that the prevalence of alcohol use by females in India, and consequent alcohol dependence is exceedingly low. Having a non-drinking or low-drinking control sample, would have lent greater depth to these findings.

Nevertheless, this study highlights the ubiquitousness of sexual problems in the heavy-drinking population. It also stresses the need for addiction medicine specialists to note the possibility of sexual problems in their clients. In addition, it highlights the need for sexual medicine specialists to consider the effects of heavy alcohol use on sexual functioning. However, there is ample evidence that alcohol-induced sexual dysfunction, for the most part, is reversible with cessation of alcohol use.[18] Thus, this information can be used in motivational counselling of heavy drinkers to provide impetus for change. Clinicians are well advised to routinely assess sexual functioning in patients with alcohol dependence.

## Appendix A

### Sexual dysfunction checklist

**Table:**

In the past 12 months…	Yes = 1		No = 0
	Global	Situational	
Has the prospect of having sex with your partner produced such aversion, fear or anxiety that you avoided any sexual activity (Aversion to sex)? Have you had a lack or loss of sexual desire, which has led you to initiating sexual activity with your partner or engaging in solitary masturbation, at a frequency clearly lower than previous levels (Low sexual desire)? Have you had a difficulty in getting an erection sufficient for intercourse (Difficulty achieving erection)? Have you had difficulty in maintaining erection (full erection occurs during the early stages of lovemaking but disappears when intercourse is attempted or before ejaculation if it occurs) (Difficulty maintaining erection)? Have you found that frequently (or always) you have ejaculated before you would like to: before entry? < 1 min? < 3 min? (Premature ejaculation) Have you frequently had problems in ejaculating even when erect (Inhibited / delayed ejaculation)? Have you ever ejaculated without getting full erection (Orgasm with flaccid penis)? Do you have difficulty in reaching the peak of pleasure at the time of ejaculation (Anorgasmia)? Have you felt pain in the genitals during sexual intercourse (Coital pain)? Are you satisfied with the frequency of sex (Frequency dissatisfaction)? Are you satisfied with your sexual relationship with your partner (Dissatisfaction of sexual relation with partner)? Are you satisfied with your sexual performance (Dissatisfaction with own sexual function)?		
